# Vaping of Cannabis, Cannabidiol, and Synthetic Cannabis Among US Sexual Minority Youths

**DOI:** 10.1001/jamanetworkopen.2023.29041

**Published:** 2023-08-15

**Authors:** Jessica Liu, Andy S. L. Tan, Juhan Lee

**Affiliations:** 1 Research and Education to Empower Adolescents and Young Adults to Choose Health (REACH) Lab, Division of Adolescent Medicine, Department of Pediatrics, Stanford University, Palo Alto, California; 2 University of Pennsylvania, Annenberg School for Communication, Philadelphia; 3 University of Pennsylvania, Leonard Davis Institute of Health Economics, Philadelphia; 4 Abramson Cancer Center, Tobacco and Environmental Carcinogenesis Program, Philadelphia, Pennsylvania; 5 Department of Psychiatry, Yale University School of Medicine, New Haven

## Abstract

This cross-sectional study investigates rates of vaping of cannabis, cannabidiol, and synthetic cannabis among US sexual minority youths.

## Introduction

Sexual minority youths, compared with heterosexual youths, reported higher past 30-day use in 2021 of alcohol (29.3% vs 21.6%), cannabis (25.6% vs 14.0%), e-cigarettes (26.2% vs 16.4%), and other tobacco (9.1% vs 4.6%).^1^ Higher rates of e-cigarette use among sexual minority youths may be associated with increased risk for vaping cannabis products.^2^ In particular, the 2019 e-cigarette, or vaping, product use–associated lung injury (EVALI) epidemic highlighted concerns of vaping illicit tetrahydrocannabinol (THC) oils.^2^ Thus, this study aimed to examine the rate of vaping cannabis among sexual minority youths.

## Methods

This cross-sectional study used the 2022 National Youth Tobacco Survey (NYTS), a Centers for Disease Control and Prevention–collected data set on tobacco use behaviors among 28 291 US nationally representative middle and high school students (weighted response rate, 45.2%). Outcomes included past 30-day vaped (1) marijuana, including THC, THC concentrates, hash oil, or waxes; (2) cannabidiol (CBD) or CBD oils; and (3) synthetic marijuana or cannabinoids, such as K2 or spice. The factor of interest was sexual identity, which was assessed as “heterosexual (straight),” “lesbian or gay,” “bisexual,” and “not sure.” Covariates included school level, sex, race and ethnicity, past 30-day nicotine vaping and other tobacco use, familial e-cigarette use, and peer e-cigarette use (all self-reported). We conducted descriptive statistics for the population count and prevalence of cannabis vaping outcomes by sexual orientation from probability weights and assessed bivariate associations with Pearson χ^2^ test (Rao and Scott correction) and ran post hoc tests for differences between sexual orientation subgroups. We further ran a series of multivariable logistic regression models on the 3 vaping outcomes by sexual orientation, adjusting for covariates, and stratified by sex. We ran multivariable models interacting sexual orientation and race and ethnicity to explore the association of that intersectionality with outcomes. We considered *P* = .0167 (.05/3) as the level of statistical significance to adjust for multiple comparisons and followed the Strengthening the Reporting of Observational Studies in Epidemiology (STROBE) reporting guidelines. We used the survey package in R statistical software version 1.1.456 (R Project for Statistical Computing) for analyses. This secondary data analysis of publicly available, deidentified data was deemed exempt from review by the Yale Institutional Review Board.

## Results

Among 28 291 students (12.4% non-Hispanic Black, 26.9% Hispanic, 54.1% non-Hispanic White, and 6.6% non-Hispanic other race; 51.1% male; 56.2% high school students), 4.5% students identified as gay or lesbian, 11.8% as bisexual, and 9.9% as “not sure.” Among males and females, sexual minority youths (gay or lesbian and bisexual individuals) reported higher levels of past 30-day vaping of THC, CBD, and synthetic cannabis than their heterosexual peers (Table 1). Post hoc tests showed that there were significant differences between gay and lesbian youths and the not sure group for all 3 vaping outcomes and for bisexual youths and the not sure group for THC and CBD vaping. After adjusting for other covariates, bisexual youths were more likely vs heterosexual youths to report past 30-day THC vaping (adjusted odds ratio [aOR] = 1.61; 95% CI, 1.24-2.09) and CBD vaping (aOR, 1.99; 95% CI = 1.37-2.91) (Table 2). We found similar patterns when stratified by sex. There was no interaction between sexual orientation and race and ethnicity for any outcome for the overall sample or when stratified by sex.

**Table 1.** Respondent Characteristics

Characteristic	Overall (N = 28 291)		Heterosexual (n = 18 740 [73.8%])^a^		Gay and lesbian (n = 1146 [4.5%])^a^		Bisexual (n = 3002; 11.8%)^a^		Not sure (n = 2547; 9.9%)^a^		χ^2^^b^
	Unweighted No.	Weighted %	Unweighted No.	Weighted %	Unweighted No.	Weighted %	Unweighted No.	Weighted %	Unweighted No.	Weighted %	
Past 30-d cannabis vaping^c^											
Marijuana^d^	2273	8.2	1291	7.2	142	14.4	387	12.8	134	0.9	<0.001
CBD or CBD oils	841	2.9	425	2.2	62	6.4	150	4.8	66	2.3	<0.001
Synthetic marijuana or cannabinoids^e^	402	1.4	191	1.0	41	4.4	60	2.1	33	1.1	<0.001
Sex											
Male	14 375	51.1	10 801	57.6	426	39.2	558	19.1	1040	40.4	<0.001
Female	13 692	48.9	7907	42.4	700	60.8	2393	80.9	1473	59.6	
Grade level											
Middle school	12 041	43.8	7541	41.2	457	37.9	1274	41.9	1461	58.2	<0.001
High school	16 118	56.2	11 172	58.8	679	62.1	1722	58.1	1075	41.8	
Race and ethnicity^f^											
Hispanic	7458	26.9	4707	25.2	308	28.8	816	28.0	722	27.7	0.103
Non-Hispanic Black	3383	12.4	2195	11.9	150	13.8	350	11.7	274	10.6	
Non-Hispanic White	13 501	54.1	9413	56.0	567	51.5	1466	54.5	1094	53.1	
Non-Hispanic other^g^	3171	6.6	2089	6.8	99	5.9	312	5.8	367	8.6	
Past-30-d other tobacco use^h^											
None	25 950	94.8	17 794	95.8	1007	87.2	2749	93.4	2416	96.6	<0.001
Any	1569	5.2	830	4.2	125	12.8	221	6.6	100	3.4	
Past 30-d nicotine vaping											
None	25 801	92.6	17 340	93.2	1014	89.2	2640	90.8	2428	95.9	<0.001
Any	2070	7.4	1236	6.8	115	10.8	297	9.2	98	4.1	
Household e-cigarette use											
No	21 377	86.5	15 731	88.0	858	77.3	2270	79.1	2145	88.1	<0.001
Yes	3338	13.5	2167	12.0	232	22.7	612	20.9	285	11.9	
Perceived peer e-cigarette use											
None	4904	18.8	3490	18.1	180	13.5	392	12.0	843	31.2	<0.001
Any	21 600	81.2	15 242	81.9	966	86.5	2610	88.0	1704	68.8	

Abbreviation: CBD, cannabidiol.

^a^ Sexual identity was assessed with the question “Which of the following best describes you?” with responses of “heterosexual (straight),” “lesbian or gay,” “bisexual,” and “not sure.” Sexual identity totals to 25 435 due to missing values.

^b^ χ^2^ values were obtained through Pearson χ^2^ test with Rao and Scott correction. Post hoc tests were conducted.

^c^ Past-30-day vaping cannabis was assessed with the questions “Have you vaped any of the following substances during the past 30 days?” and separate items for “marijuana (also called pot, weed, or cannabis), including THC, THC concentrates, hash oil, or waxes,” “CBD or CBD oils,” and “synthetic marijuana or cannabinoids, such as K2 or spice” (not mutually exclusive). Totals for past-30-day cannabis across sexual identity groups do not total to overall due to missing values from sexual identity.

^d^ Marijuana (also called pot, weed, or cannabis), includes THC, THC concentrates, hash oil, or waxes.

^e^ Includes as K2 or spice.

^f^ Available responses for race were American Indian or Alaska Native, Asian, Black or African American, Native Hawaiian or Other Pacific Islander, and White. Available responses for ethnicity were Hispanic, Latino, Latina, or of Spanish origin (Mexican, Mexican American, Chicano, or Chicana; Puerto Rican; Cuban; and another Hispanic, Latino, Latina, or Spanish origin) or not of Hispanic, Latino, Latina, or Spanish origin. Race and ethnicity were asked in separate questions and combined by ignoring race responses from individuals who responded yes to Hispanic, Latino, Latina, or of Spanish origin.

^g^ Non-Hispanic other includes non-Hispanic Asian, non-Hispanic American Indian or Alaska Native, non-Hispanic Native Hawaiian or Other Pacific Islander, and multiple non-Hispanic races (respondents could choose more than 1 listed race).

^h^ Other tobacco use includes combustible cigarettes, cigars or cigarillos, smokeless tobacco, hookahs, roll-your-own cigarettes, pipes, snus, dissolvable tobacco, bidis, heated tobacco products, and nicotine pouches (does not include nicotine vaping).

**Table 2.** Association of Sexual Identity With Cannabis Vaping Outcomes

Sexual identity	Past 30-d THC vaping^a^		Past 30-d CBD vaping^a^		Past 30-d synthetic cannabis vaping^a^	
	aOR (95% CI)	*P* value^b^	aOR (95% CI)	*P* value^b^	aOR (95% CI)	*P* value^b^
**Overall (N = 28 291)**						
Sexual orientation						
Heterosexual	1 [Reference]	NA	1 [Reference]	NA	1 [Reference]	NA
Gay or lesbian	1.56 (1.01-2.41)	.04	1.87 (0.96-3.65)	.07	2.26 (0.91-5.60)	.08
Bisexual	1.61 (1.24-2.09)	<.001	1.99 (1.37-2.91)	<.001	1.83 (0.90-3.75)	.10
Not sure	1.23 (0.84-1.78)	.28	1.64 (0.97-2.79)	.07	1.90 (1.03-3.50)	.04
**Males (n = 14 375)**						
Sexual orientation						
Heterosexual	1 [Reference]	NA	1 [Reference]	NA	1 [Reference]	NA
Gay or lesbian	1.74 (0.78-3.90)	.18	2.50 (0.81-7.70)	.11	3.81 (1.19-12.20)	.03
Bisexual	2.13 (1.35-3.36)	.001	3.67 (1.65-8.17)	.002	3.10 (1.11-8.67)	.03
Not sure	1.38 (0.68-2.81)	.27	1.83 (0.82-4.08)	.14	2.14 (0.90-5.08)	.08
**Females (n = 13 692)**						
Sexual orientation						
Heterosexual	1 [Reference]	NA	1 [Reference]	NA	1 [Reference]	NA
Gay or lesbian	1.42 (0.89-2.28)	.14	1.51 (0.75-3.05)	.25	1.29 (0.48-3.48)	.61
Bisexual	1.43 (1.02-2.01)	.04	1.56 (1.12-2.18)	.009	1.38 (0.66-2.88)	.39
Not sure	1.13 (0.67-1.90)	.66	1.53 (0.74-3.17)	.25	1.55 (0.67-3.60)	.31

Abbreviations: aOR, adjusted odds ratio; CBD, cannabidiol; NA, not applicable; THC, tetrahydrocannabinol.

^a^ Models were adjusted for grade level (middle or high school), sex (male or female), race and ethnicity (Hispanic, non-Hispanic Black, non-Hispanic White, and non-Hispanic other), any past 30-day use of other tobacco (none or any), household e-cigarette use (yes or no), any past 30-day nicotine vaping (none or any), and perceived peer e-cigarette use (none or any).

^b^ The statistical cutoff of the *P* value was .016 (.05/3), adjusted for multiple comparisons.

## Discussion

In this cross-sectional study, sexual minority youths, particularly bisexual youths, were more likely to vape THC and CBD, as was found in a California sample.^3^ It is well-documented that minority stress (eg, stress from sexual orientation–based discrimination) is associated with youth substance use,^4^ which may be consistent with vaping cannabis.

This study has several limitations, such as self-report bias and an observational, cross-sectional nature, which cannot establish causal inference. This study did not assess dose and frequency. There may be unmeasured confounders, such as alcohol and other substance use, which the NYTS does not include. Despite these limitations, preliminary evidence from this study may inform future prevention strategies directed at reducing substance use disparities among sexual minority youth.

## Notes

Supplement.
Data Sharing Statement
