# Supplementary material for: Vaping of Cannabis, Cannabidiol, and Synthetic Cannabis Among US Sexual Minority Youths
Source: JAMA Netw Open. 2023 Aug 15;6(8):e2329041. doi: 10.1001/jamanetworkopen.2023.29041 (PMC10427937; doi:10.1001/jamanetworkopen.2023.29041)
Supplement: Supplement. — Data Sharing Statement [file jamanetwopen-e2329041-s001.pdf]

Data Sharing Statement

Liu. Vaping of Cannabis, Cannabidiol, and Synthetic Cannabis Among US Sexual Minority Youths. *JAMA Netw Open*. Published online August 15, 2023. doi: 10.1001/jamanetworkopen.2023.29041

Data
Data available: Yes
Data types: Deidentified participant data
How to access data: https://www.cdc.gov/tobacco/data_statistics/surveys/nyts/data/index.html
When available: With publication

Supporting Documents
Document types: None

Additional Information
Who can access the data: Anyone requesting the data
Types of analyses: For any purpose
Mechanisms of data availability: With investigator support
